# Oleogel-S10 Phase 3 study “EASE” for epidermolysis bullosa: study design and rationale

**DOI:** 10.1186/s13063-019-3362-z

**Published:** 2019-06-11

**Authors:** Johannes S. Kern, Agnes Schwieger-Briel, Sandra Löwe, Mark Sumeray, Charles Davis, Anna E. Martinez

**Affiliations:** 1 Dermatology Department, Royal Melbourne Hospital, Faculy of Medicine, Dentistry and Health Science, The University of Melbourne, Parkville, Victoria Australia; 2 Department of Dermatology, Faculty of Medicine, Medical Center - University of Freiburg, Freiburg, Germany; 3 Department of Dermatology, University Childrens’ Hospital Zurich, Zurich, Switzerland; 4 Amryt Pharmaceuticals DAC, Dublin, Ireland; 5 CSD Biostatistics Inc., Oro Valley, AZ USA; 6 Department of Paediatric Dermatology, Great Ormond Street Hospital NHS Foundation Trust, London, UK

**Keywords:** Epidermolysis bullosa, Kindler syndrome, Oleogel, Birch bark, Betulin, Trial design, Wound healing

## Abstract

**Background:**

Epidermolysis bullosa (EB) is a group of rare, genetic diseases that affect the integrity of epithelial tissues, most notably the skin. Patients experience recurrent skin wounding, with severity depending on type, sub-type, and mutation. Oleogel-S10, a formulation of birch bark extract, has demonstrated efficacy in a Phase 2 trial assessing re-epithelialization of wounds in EB. EASE (NCT03068780, EudraCT 2016–002066-32) is a randomized, Phase 3, placebo-controlled study designed to determine the efficacy of Oleogel-S10 versus placebo in patients with EB.

**Methods:**

EASE is a Phase 3, two-phase study comprising a 90-day, double-blind, randomized, placebo-controlled phase, followed by 24 months of open-label, single-arm follow-up. Patients with junctional EB, dystrophic EB, or Kindler syndrome and target wounds (10 - 50cm^2^) present for > 21 days and < 9 months, are randomized in a 1:1 ratio to receive wound dressings according to local standard of care with or without Oleogel-S10. Placebo is based on the Oleogel-S10 vehicle, which is sunflower oil formulated to have a consistency indistinguishable from that of the active product. The primary endpoint of the trial, directed by the US health authority according to the required study endpoints for chronic cutaneous ulcer and burn wounds, is to compare the efficacy of Oleogel-S10 versus placebo according to the proportion of patients with complete closure of the target wound within 45 ± 7 days of treatment. Additional EB-focused endpoints include wound burden, patient-reported outcomes, and safety.

**Results:**

Results of the primary endpoint are anticipated to be available by H2 2019.

**Trial registration:**

ClinicalTrials.gov, NCT03068780. EudraCT, 2016–002066-32. Registered on 3 March 2017.

**Electronic supplementary material:**

The online version of this article (10.1186/s13063-019-3362-z) contains supplementary material, which is available to authorized users.

## Background

Epidermolysis bullosa (EB) is a rare group of autosomal, dominant or recessive, genetic disorders characterized by mechanical fragility of skin and mucosal surfaces. In EB, the skin blisters in response to minor trauma or friction [1]. There are > 30 genetically and phenotypically distinct types and sub-types of EB caused by mutations in approximately 20 genes. Classification of EB is made according to cleavage level within the skin ultrastructure, particular morphological features, the causative gene, and the inheritance pattern [1].

Different sub-types of EB are characterized by differing extents of extra-cutaneous involvement, including many organs such as the gastro-intestinal and urinary tract, larynx, eyes, bones, etc. These secondary complications are mainly common in dystrophic (DEB) and junctional EB (JEB) and less common in EB simplex (EBS) [1]. Most EB patients with the less severe forms of EB have a normal life expectancy; however, severe forms, which are generally evident in infancy, are life-limiting with death resulting from infection, sepsis, failure to thrive, or squamous cell carcinoma (SCC) [1].

There is currently no cure for any form of EB. Management is centered on wound care and prevention, or on early treatment of complications as far as is possible. In addition, patients with specific sub-types of EB also need to be monitored for extra-cutaneous manifestations such as corneal abrasions, dysphagia, osteoporosis, pseudosyndactyly, and development of SCC [1]. The procedures for wound care are meticulous, time-consuming, and often very painful for patients. They also result in a great burden for the patients and their families.

In common with other rare genetic diseases, robust research activities are ongoing into the development of recombinant genetic and stem cell therapies for EB. However, these experimental approaches have not yet resulted in viable routine therapeutic treatment options [2]. By definition, gene therapy for EB is directed towards repair of the erroneous DNA coding for the non-functioning protein; therefore, each genotype requires a separate gene- or protein-directed therapy.

Oleogel-S10 is an herbal preparation made from dry birch bark extract (also referred to as triterpene extract [TE]) combined with sunflower oil. Oleogel-S10 is formulated as dry extract from birch bark: 10 mg dry extract from birch bark and 90 mg refined sunflower oil per 100 mg of product. Betulin comprises 72 to 88% of the birch bark extract. The other major marker substances include betulinic acid (0.5–6%), lupeol (2–8%), oleanolic acid (0.1–2%), and erythrodiol (0.5–2%) [3]. TE has activity on keratinocytes at various stages throughout the wound-healing process, including modulation of inflammatory mediators, and stimulation of keratinocyte migration and differentiation [4, 5]. Other components of birch bark extract besides betulin (e.g. oleanolic acid, erythrodiol, betulinic acid, and lupeol) also have anti-inflammatory effects [6].

Oleogel-S10 was studied in a clinical trial program including Phase 2 studies—one in split-thickness skin grafts (STSG) and one in EB [7]—as well as Phase 3 studies in burns and STSG [8]. Studies in STSG and burns support the development of Oleogel-S10 in EB because EB wounds share morphological characteristics of partial thickness wounds. Specifically, the level of skin cleavage in the four major sub-types of EB primarily extends, at a maximum, into the superficial dermis [9]. In a small, Phase 2 proof-of-concept trial in patients with EB (EudraCT 2010–019945-24), 12 wound pairs in 10 patients with DEB were treated with Oleogel-S10 in an open, blindly evaluated, controlled fashion. Re-epithelialization was considered to be superior for Oleogel-S10 compared with standard of care by both of two blinded reviewers in five of the 12 cases. In three cases, only one reviewer regarded Oleogel-S10-treated wounds to have better re-epithelialization than standard of care [7]. The relatively small size of this trial means that firm conclusions on the efficacy of Oleogel-S10 in EB cannot be made; however, the low incidence of adverse events (AEs), and the possibility of improved efficacy over standard of care, indicated that a Phase 3 trial was warranted. Oleogel-S10 has been approved in Europe for the treatment of partial thickness wounds in adults [3] based on data from STSG and burns studies in adults [8, 10].

EASE (Efficacy and safety of Oleogel-S10 in patients with EB) is a randomized (1:1), double-blind, placebo-controlled, superiority Phase 3 trial of Oleogel-S10 in patients with EB ; therefore, it is the pivotal trial to support the determination of the efficacy and safety of Oleogel-S10 in EB. Due to the lack of other effective pharmacological interventions in EB, EASE utilized standard-of-care wound dressing with a placebo gel as a comparator. The primary objective of the double-blind phase is to compare the efficacy of Oleogel-S10 with placebo in the promotion of healing of EB partial thickness wounds. This paper discusses the design and rationale of the EASE trial, the difficulties of designing an appropriate study in the complex setting of EB, as well as the general difficulties in designing and conducting clinical trials in rare diseases. EASE is registered as NCT03068780, EudraCT 2016–002066-32; registered on 3 March 2017; https://clinicaltrials.gov/ct2/show/NCT03068780.

## Methods

### Design

**Fig. 1:**
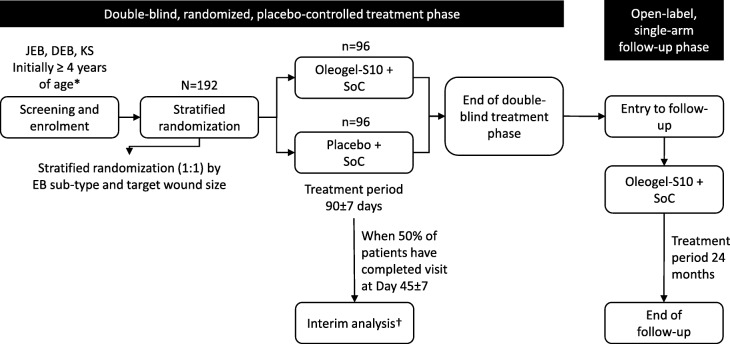
Trial design. *Children aged < 4 years may be included only after confirmation by the Independent Data Monitoring Committee (IDMC) upon review of the safety and bioanalytical data available when at least six children aged 4–11 years, plus at least the same number of older children and adults have completed days 45 and 90. †The unblinded interim analysis for sample size re-estimation will take place when approximately 50% of patients have completed day 45 ± 7. Depending on the results of the sample size re-estimation, the IDMC will recommend continuing with the initial sample size, increasing the sample size, or stopping the study for futility. DEB dystrophic epidermolysis bullosa, JEB junctional epidermolysis bullosa, KS Kindler syndrome, SoC standard of care

EASE is a two-phase study comprising a 90-day, double-blind, randomized, placebo-controlled treatment phase, followed by a 24-month, open-label, single-arm follow-up phase (Fig. 1).

In the double-blind phase, patients are randomized 1:1 to either Oleogel-S10 plus standard-of-care wound dressing or placebo, also with standard-of-care wound dressing at least once every four days.

**Fig. 2:**
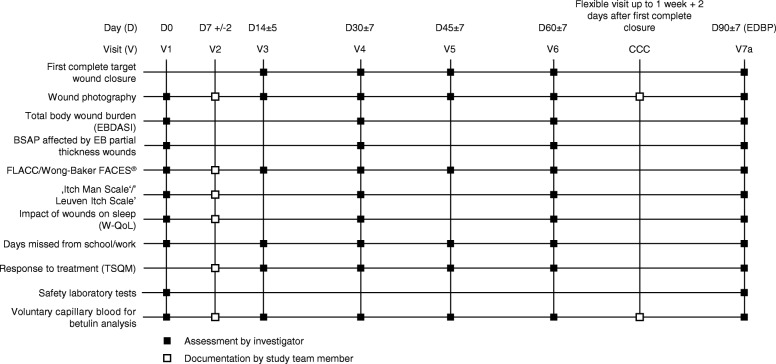
Study visit schedule. BSAP body surface area percentage, CCC confirmation of complete closure of the EB target wound, D day, EBDASI Epidermolysis Bullosa Disease Activity and Scarring Index, EDBP end of double-blind phase, FLACC Face, Legs, Activity, Cry, Consolability scale, TSQM Treatment Satisfaction Questionnaire for Medication; V visit, W-QoL Wound-Quality of Life Questionnaire

One EB target wound is assigned for each patient. The target wound must involve loss of the epidermis, with extension into the dermis allowable. The wound must be 10–50 cm^2^ in surface area and be > 21 days and < 9 months old according to the patient’s report. In the Oleogel-S10 arm, Oleogel-S10 will be applied to the target wound at the same time as the dressing changes. Data from pre-clinical and clinical studies have shown efficacy and safety within this range of dosing frequency. Oleogel-S10 gel or corresponding placebo will be administered topically to all wound areas on the body in a layer of approximately 1-mm thickness and will be covered with a non-adhesive wound dressing. This is the same thickness that was applied in previous clinical studies of Oleogel-S10 [7, 8, 11]. Standard-of-care dressing (patient or physician choice) will be non-adhesive wound dressing (e.g. soft silicone or foam) or equivalents as described in the International Consensus Best Practice Guidelines for Skin and Wound Care in Epidermolysis Bullosa [12]. In common with the Oleogel-S10 arm, wound dressings will be applied at least once every four days according to patient preference. Placebo will be applied in the same manner as for Oleogel-S10. The placebo will be sunflower oil formulated to have a consistency indistinguishable from that of Oleogel-S10. In both arms, wounds will be cleaned before application of dressings and Oleogel-S10. The schedule of study visits is shown in Fig. 2.

Randomization will be stratified according to EB sub-type. Within each sub-type, wounds will be stratified by size (10–19 cm^2^, 20–29 cm^2^, and 30–50 cm^2^). Randomization will be conducted according to blinded patient number and the randomization key will be held solely by an independent statistician. Patients are randomized by accessing the patient in the electronic trial database (RAVE) and randomizing him/her in the section ‘Randomisation’ in the electronic clinical report form (eCRF). Here, randomization date, time, number, kit number, and date of allocation are automatically populated once it is confirmed that the patient is ready for randomization and entered how many kits need to be allocated. In the event of an emergency, unblinding is conducted electronically with full documentation of the unblinding request and sample size re-estimation.

An unblinded, interim efficacy analysis and sample size re-estimation will be conducted when 50% of patients have reached day 45 ± 7. Depending on the results of the sample size re-estimation, the Independent Data Monitoring Committee (IDMC) will recommend continuing with the initial sample size, increasing the sample size, or stopping the study for futility. The IDMC will review blinded safety data when at least six children aged 4–11 years, plus at least the same number of older children and adults, have completed days 45 and 90.

During the open-label phase, Oleogel-S10 will continue to be applied in conjunction with standard-of-care wound dressings. Once the last visit for the randomized phase has been completed and the return of the corresponding unused study medication has occurred, the patient may enter the single-arm, open-label, 24-month follow-up period. The last visit on day 90 of the randomized phase corresponds to the first visit of the follow-up phase, whereby photographic data from the former will become the baseline for the latter. All patients will receive Oleogel-S10 in accordance with the method and timings used in the randomized phase. Compliance will be assessed via records of gel tube dispensing, weighing, and return of spent and unused containers.

After the end of follow-up, it is intended that patients will receive Oleogel-S10 on a named-patient basis, where feasible as per local regulation, until approval of the investigational product.

### Endpoints

The primary objective of the EASE trial is to compare the efficacy of Oleogel-S10 with placebo. The primary endpoint used to determine efficacy is the proportion of patients with first complete closure of the target EB wound within 45 ± 7 days of treatment (Table 1). The assessment for the primary endpoint follows the U.S. Food and Drug Administration (FDA) Guidance for Industry ‘*Chronic Cutaneous Ulcer and Burn Wounds – Developing Products for Treatment*’ [13]. There is no guidance on the specified time of an acute EB wound becoming chronic, although the consensus meetings on the development of the Instrument for Scoring Clinical Outcome of Research for Epidermolysis Bullosa (iscorEB) [14] have defined wounds in EB to be chronic if they are present for > 6 weeks [14]. Therefore, the closure time target of 45 days, plus the 21-day baseline wound age is in line with this chronicity (i.e. > 42 days).

**Table 1:** Primary and secondary outcome measures

Primary endpoint	• Proportion of patients with complete closure of the target wound within 45 ± 7 days of treatment
Secondary endpoints	• Time to wound closure up to 90 ± 7 days of treatment (key secondary endpoint) • Incidence of first complete wound closure of EB target wound at different time points (see Fig. 2) • Change from baseline in EB target wound size • Change in total body wound burden over time • Change in percentages of TBSA affected by EB partial thickness wounds
Patient-reported outcomes	• Change from baseline in background and procedural pain after wound dressing change • Change from baseline in itching before wound dressing change • Response to treatment • Change from baseline in sleep quality • Number of days missed from school or work
Safety endpoints	• Incidence, severity, and relatedness of AEs • Local tolerability • Laboratory findings • Incidence and severity of wound infections

*AE* adverse event, *EB* epidermolysis bullosa, *TBSA* total body surface area

The definition of wound closure for the EASE study is first appearance of complete re-epithelialization without drainage. Once the target wound is deemed closed based on clinical assessment by the Investigator, a Confirmation of Wound Closure visit will occur within 7 + 2 days. Study sites will educate patients and parents verbally and through the use of photographs as to what re-epithelization means and how to recognize this for their wounds. Patients and parents will be instructed to contact the site as soon as they believe that re-epithelization without drainage has occurred – the site will schedule the next planned study visit as soon as possible or arrange for the patient to attend an unscheduled visit for the purpose of wound assessment.

The key secondary efficacy endpoint of the double-blind phase will be the comparison in efficacy of Oleogel-S10 versus placebo according to the time to first complete closure of the EB target wound until day 90 ± 7. The time taken to achieve complete wound healing is a clinically important endpoint for the assessment of the potential benefit of a wound-healing treatment in EB. Faster wound healing results in fewer symptoms related to open wounds (e.g. pain and itching) and would be expected to decrease the likelihood of wound infection. Additional secondary endpoints are shown in Table 2, and include patient-reported outcomes (PROs) and reduction of total body surface area wound burden.

**Table 2:** Inclusion and exclusion criteria

Inclusion criteria	Exclusion criteria
Male and female patients with JEB, DEB, or Kindler syndrome aged ≥ 4 years (children aged < 4 years may be included only after confirmation by the Independent Data Monitoring Committee upon review of the safety and bioanalytical data at the interim safety review stage) Patients with an EB target wound (i.e. EB partial thickness wound of 10–50 cm^2^ in size aged ≥ 21 days and < 9 months) with no signs of local infection Patient and/or his/her legal representative has/have been informed, has/have read and understood the patient information/informed consent form, and has/have given written informed consent Patient and/or his/her legal representative must be able and willing to follow study procedures and instructions	Patients with EBS EB target wound with clinical signs of local infection Use of systemic antibiotics for wound-related infections within 7 days before enrollment Administration of systemic or topical steroids (except for inhaled, ophthalmic, or topical applications, such as budesonide suspension for esophageal strictures [e.g. Pulmicort Respules® 0.25 mg/2 mL or 0.5 mg/2 mL]) within 30 days before enrollment Immunosuppressive therapy or cytotoxic chemotherapy within 60 days before enrollment Patient has undergone stem cell transplant or gene therapy for the treatment of inherited EB Current and/or former malignancy including basal cell carcinomas and squamous cell carcinomas Enrollment in any interventional study or treated with any investigational drug for any disease within 4 weeks before study entry Factors present in the patient and/or his/her legal representative that could interfere with study compliance such as inability to attend scheduled study visits or compliance with home dressing changes Pregnant or nursing women Women of childbearing potential including post-menarchal female adolescents and men who are not willing to use an effective form of birth control with failure rates < 1% per year (e.g. implant, injectable, combined oral contraceptive, intrauterine contraceptive device, sexual abstinence, vasectomy, or vasectomized partner) during participation in the study (and at least 3 months thereafter) Patient is a member of the investigational team or his/her immediate family Patient lives in the same household as a study participant

*EB* epidermolysis bullosa, *DEB* dystrophic EB, *EBS* EB simplex, *JEB* junctional EB

The primary rationale of the open-label follow-up is to obtain long-term safety data, but efficacy data will continue to be collected according to the methods of the double-blind phase.

For the assessment of wound closure and re-epithelialization, the investigator will photograph the EB target wound and all other wounds that match target wound criteria with the ARANZ Silhouette® system. This system measures accurately, precisely, and reliably, provides high quality imaging, and a standardized documentation. The system consists of the SilhouetteStar™ point of care imaging device that captures the wound image using three-dimensional (3D) laser technology and SilhouetteConnect™ software that creates a 3D model of the wound based on photographic data, derives measurements of the model, and records standardized notes. Automatic flash ensures consistent lighting across images.

During screening, the investigator will select the EB target wound and two appropriate anatomical landmarks on either side of it. The baseline reference image will be taken with these landmarks. Future visits will refer to the baseline reference image to ensure that the correct wound is assessed. All other wounds that match target wound criteria will be photo-documented similarly.

Post-treatment assessments will be made within one week of wound closure to determine durability of healing. This one-week window was selected as an adaptation of an FDA requirement for confirmatory assessment of wound closure two weeks after first determination, as used for wounds such as diabetic ulcers. For the EASE trial, the two-week confirmatory assessment was reduced to one week because of the tendency of EB wounds to re-open per the normal course of the disease state. This FDA requirement poses a major challenge for reaching meaningful results in the context of EB, where re-wounding is a common occurrence [15]. Data collection forms for wound assessment are provided in the protocol. These forms are part of an overall eCRF that documents all patient data from screening, through baseline, treatment, and follow-up. The eCRF is completed by the investigator and is subject to a data management procedure (documented separately from the protocol) that included review and query of errant data. A contract coder will code any AEs according to MedDRA and any concomitant medications according to the World Health Organization Drug Dictionary.

Safety will be assessed by the overall incidence, severity, and relatedness of AEs. These will be captured via the eCRF and subject to the same data management and query procedure as the other endpoints.

PROs will be assessed according to the Itch Man Scale [16], the Leuven Itch Scale [17], the Face, Legs, Activity, Cry, Consolability Pain Rating Scale [18], the Wong-Baker FACES® Pain Rating Scale [19], the impact of wounds on sleep quality [20], Treatment Satisfaction Questionnaire for Medication [21], and number of days missed from school or work. These endpoints will be applied according to age category. Data collection forms for PROs are provided in the protocol.

For the open-label, follow-up phase, key endpoints are incidence and severity of AEs, local tolerability, and laboratory-related safety data. Efficacy endpoints from the randomized trial will also be evaluated.

Appropriately trained monitors will periodically contact the site and perform site visits in accordance with applicable regulations, Good Clinical Practice, and sponsor-approved procedures.

### Patients

EASE will enroll male and female patients aged ≥ 4 years with JEB, DEB, or Kindler syndrome. EASE will not enroll patients with EBS because this sub-type of EB often only has a mild phenotype with minor blisters [12, 22] and hence would be the least likely sub-type of EB to derive a treatment benefit; therefore, inclusion of EBS patients would be likely to dilute the overall treatment effect. Exclusion of EBS patients will help to ensure that healing rates in the control arm of the study will not be too high and thereby avoid reduction in statistical power while increasing the likelihood of demonstrating a statistically significant treatment effect in other treatment groups. Detailed inclusion and exclusion criteria are given in Table 2. Children aged < 4 years may be included only after confirmation by the IDMC upon review of the safety and bioanalytical data at the interim safety review stage. The IDMC will evaluate safety parameters, including any off-target, systemic effects of Oleogel-S10. Additional patients aged < 4 years will need to be ≥ 21 days old because of the need for EB wounds to be ≥ 21 days in evidence.

Recruitment and retainment are aided by patient brochures, an informed consent flipchart, a study flowchart, a dosing guide, a booklet for children, an emergency information card, and cards that cover inclusion and exclusion criteria.

**Fig. 3:**
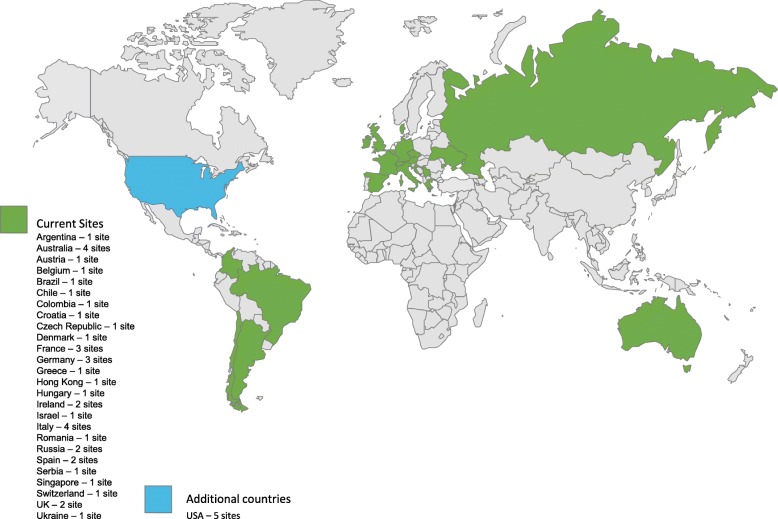
Study locations. Data correct July 2018

EASE is conducted in multiple countries (Fig. 3) in an outpatient, home-care setting in accordance with the principles of the Declaration of Helsinki. The trial has received institutional review board approval at all participating sites. All patients/guardians provide written informed consent before enrollment.

Patients will be expected to complete the randomized phase before enrollment in the open-label phase. However, if a patient discontinues the randomized phase prematurely due to worsening of the EB target wound status or due to EB target wound infection, the investigator may decide to allow the patient to enter the follow-up phase prematurely or else discontinue the study.

Until complete closure and confirmed epithelialization during both the double-blind phase and follow-up phase of the study, patients will not be permitted to use sulfadiazine silver, silver dressing, topical antibiotics, or topical steroids for the treatment of study target wound areas and other wounds matching target wound criteria, as these products have the potential to either impact wound healing or introduce assessment bias in photographic wound area measurement/assessment. These treatments will be allowed on single, non-target EB wounds. Application of creams and ointments on areas on the patient’s body that are affected by EB wounds will not be permitted during the double-blind phase of the study. Until M3 ± 14 days of the open-label follow-up, systemic steroids - except for inhaled, ophthalmic, or topical applications, such as budesonide suspension for esophageal strictures (e.g. Pulmicort Respules® 0.25 mg/2 mL or 0.5 mg/2 mL) - and immunosuppressives are not permitted, nor is it allowed to use systemic antibiotics with the expressed purpose of reducing EB target wound area.

### Statistical considerations

The sample size estimation assumed a true control rate for the primary endpoint of 27% based on estimation of the expected wound-healing rate in the control arm of EASE, which in turn was calculated using wound-healing rates from the ESSENCE trial of allantoin cream in EB. Based on a two-sided two-sample comparison of proportions at the alpha = 0.05 level of significance, a sample size of 91 patients in each group (total 182) will provide 80% power to detect an improvement of 20 percentage points (i.e. a rate of 47% in the Oleogel-S10 arm). To account for an estimated drop-out rate of 5%, 192 patients are planned to be enrolled (96 patients per arm).

For the primary endpoint, the proportion of patients with first complete closure of the EB target wound within 45 ± 7 days based on clinical assessment by the investigator in the Oleogel-S10 and placebo treatment groups will be compared using the Cochran-Mantel-Haenszel (CMH) test, stratified by EB sub-type and target wound size class. Due to the interim analysis, the final statistical analyses of the primary endpoint will be performed based on the Cui, Hung, Wang approach using a weighted statistic [23].

If the primary efficacy endpoint demonstrates superiority of Oleogel-S10 at the two-sided 5% significance level then the key secondary endpoint, time to first wound closure, will be tested at the two-sided 5% significance level using the non-stratified log-rank test. This hierarchical testing strategy ensures that the overall significance level remains at 5% without the need for adjustment due to multiple comparisons. Additionally, the stratified log-rank test, with consideration of EB sub-types as strata, will be conducted on the full analysis set. Further potential risk factors will be investigated by a Cox regression model on full analysis set with adjustment for EB sub-types, target wound size class, and additional baseline factors.

The secondary endpoint of the proportion of patients with first complete closure of the EB target wound will be analyzed in the same manner as the primary endpoint. The percentage change from baseline in EB target wound size will be analyzed at each visit using an analysis of covariance (ANCOVA) model including treatment group and EB sub-type as fixed effects and size of target wound at baseline as a covariate. The 95% confidence intervals for the difference in least squares means between treatment groups will be calculated. Additionally, treatments will be compared using a two-sided Wilcoxon Rank Sum test stratified by EB sub-type (van Elteren test). A sensitivity analysis will be performed using the assessment of all photographs of EB partial thickness wounds. The changes from baseline in total body wound burden, in body surface area percentage (BSAP) of total body surface area (TBSA) affected by EB partial thickness wounds, in the impact of wounds on sleep, and the treatment response will be analyzed correspondingly. The incidence rates of wound infection between treatments will be compared using a CMH test considering the strata of EB sub-type and target wound size class. Maximum severity will be compared between treatments using a two-sided Wilcoxon Rank Sum test stratified by EB sub-type and target wound size class (van Elteren test). The change from baseline in “background pain,” “procedural pain,” and itching will also be compared using a two-sided Wilcoxon Rank Sum test stratified by EB sub-type and target wound size class (van Elteren test).

For the primary endpoint, an individual with missing data will be defined as not having achieved complete closure. For the key secondary endpoint, participants will be censored at the date last known to have not achieved complete closure. Missing data for all other endpoints will be imputed according to last observation carried forward.

### Termination criteria

Patients will be withdrawn from the study if they meet any of the following criteria: worsening of the EB target wound status or EB target wound infection as assessed by the investigator (optional, as per decision of the investigator); withdrawal of patient’s and/or his/her legal representatives consent; patient is non-compliant with the study procedures or medications in the opinion of the investigator; progression of a medical condition, which, in the opinion of the investigator, should preclude further participation of the patient in the study; administration of non-permitted concomitant medication(s); investigator’s decision that a change of therapy is in the patient’s best interest; pregnancy as evidenced by a positive pregnancy test; or occurrence of an AE, which makes discontinuation desirable or necessary in the investigator’s and/or the patient’s opinion.

### Study locations

EASE is an international study conducted in 22 countries, with a further four countries in a possible expansion (Fig. 3). Sites in the United States will join the trial following approval of the protocol by the FDA.

## Results

The first patient was enrolled in EASE in the first quarter of 2017. The interim analysis conducted by the IDMC recommended that the trial should continue with an increase of 48 patients in the study to a total of 230 evaluable patients in order to achieve 80% statistical power. The analysis was conducted using unblinded efficacy data received by the IDMC for the primary endpoint from the first half of the study. A safety analysis conducted by the IDMC of data of all visits performed up to 31 December 2018 recommended to expand the inclusion of children with EB to all ages, i.e. ≥ 21 days and < 4 years per protocol. The study is predicted to complete enrollment in Quarter 3 of 2019. The last patient is expected to reach day 90 in Quarter 4 of 2019. EASE is expected to provide results from its complete dataset Quarter 4 of 2019. Results from EASE will be published and summary data will be made available on EudraCT and ClinicalTrials.gov. Publications will be prepared in accordance with Good Publication Practice and the principles set out by the International Committee of Medical Journal Editors. Editorial support may be sought.

## Discussion

The EASE trial is designed as the pivotal study forming the basis of regulatory submissions for approval of the use of Oleogel-S10 in combination with standard of care to accelerate wound healing in patients with EB. The trial has an inclusive design that includes all types of inherited EB except EBS and includes all patients aged ≥ 4 years, with expansion into younger ages based on protocol amendment 5 reflecting the IDMC decision after the unblinded interim safety analysis.

Oleogel-S10, a formulation of dry extract from birch that modulates inflammatory mediators and stimulates keratinocyte migration and differentiation [4, 5], is expected to show efficacy in EB due to its demonstrated efficacy on morphologically similar STSG and burn wounds. The drug has also shown wound-healing activity in a Phase 2 trial in EB where it has also been associated with good levels of safety and tolerability.

The EASE trial is faced with a range of issues that are common to the design of other trials for wound-healing interventions and that challenge the convention on how clinical trials ought to be designed. In general, evidence-based medicine demands the highest quality data from randomized, controlled trials. The use of standard-of-care dressings means that local study site practices can be used, which enables the trial to accurately reflect real clinical settings [24].

### Primary endpoint

The selection of the primary endpoint is an important consideration of the EASE trial. Difficulties in selecting endpoints in wound-healing trials have been encountered in the past. The recent ESSENCE trial (NCT02384460), which evaluated the efficacy of SD-101 6% allantoin cream, utilized two primary endpoints of time to complete target wound closure (within three months) and the proportion of patients experiencing complete closure of their target wound (also within three months). In that study, 49% of patients on SD-101 experienced wound closure within the three-month treatment period compared to 54% of the placebo patients (data in press reports only [25]). Thus, the data did not demonstrate evidence of a treatment effect and development of SD-101 was cancelled.

For the EASE trial, the sponsor is obliged to select a primary endpoint derived from historical trials of wound-healing therapies. In these trials, the endpoints are based on complete healing of the selected target wound and a comparison of how many closures occur in a given timeframe. These stipulations are governed by health authorities [13]. In its guidance, the FDA has listed four different kinds of endpoints that are acceptable for deriving clinical benefit in wound healing. These include: incidence of complete wound closure; speed of wound closure; facilitation of surgical wound closure; and “quality of healing,” which encompasses cosmesis and skin function [13]. However, in EB, these endpoints generate a number of problems. For incidence and speed of complete wound closure, the relapsing, remitting course of EB means that some wounds never fully close. Facilitation of surgical closure is not relevant to EB because of the wide area of involvement whereby wounds resemble partial thickness wounds rather than incisions. In a recent study of patients with recessive DEB (RDEB), Solis et al. examined the size and chronicity of wounds in RDEB. Their cross-sectional survey found that patients with the condition have chronic wounds that can persist for years and that scars form with frequent healing and reblistering within weeks [26]. Two types of wounds were identified—recurrent and chronic open [26]—and, by nature, neither of these are suited to assessment by the four criteria set out by the FDA. Delegates from the Dystrophic Epidermolysis Bullosa Research Association (DEBRA) USA met with the FDA in April 2018 to discuss pathways to drug approval; a report from that meeting is pending.

Many of the themes that were discussed at the DEBRA meeting were addressed in the new FDA guidance [27]. This guidance has indicated that clinical trials in EB are receiving special attention that differentiates them from clinical trials in burns and other wound types [27]. Notably, the draft guidance suggests that clinically meaningful improvements may involve the improvement of only one symptom or sign of EB and may require only one well-controlled trial to demonstrate efficacy depending on the persuasiveness of the data. Among the symptoms considered by the FDA are significant relief from itching, pain, blister prevention, and wound healing. The FDA suggests that PROs ought to be included in clinical trials in EB, but that findings of that nature would not be definitive for determining efficacy.

Other drug manufacturers have looked at different endpoints in EB. In a recent Phase 2/3, randomized, crossover, placebo-controlled trial of diacerein cream in EBS (Castle Creek), the primary endpoint was the proportion of patients with a reduction of > 40% from baseline in number of blisters within the treated areas through the end of each four-week treatment episode [28]. Among the secondary endpoints was the proportion of patients with recurrence of initial blister numbers plus or minus 10% at the end of both treatment periods. The study met its primary endpoint for 85% of patients in the first treatment period [28]. However, it is important to bear in mind that EBS wounds do not open in the same way that wounds open in DEB, JEB, and Kindler syndrome [22]. A further trial of diacerein (NCT03154333) used the primary endpoint as the proportion of participants who achieve ≥ 40% reduction in total area affected by EBS lesions. This is in line with the idea of measuring wound burden in EB rather than wound closure.

### Secondary endpoints: patient-reported outcomes

The current battery of assessments for wound healing may ignore the types of improvement that are most valued by patients and physicians. Frew et al. have developed a quality-of-life questionnaire specific to EB. As part of the generation of the instrument, researchers conducted interviews with 26 EB patients, 33 family members, and 11 healthcare professionals concerned with EB care [29]. In developing their instrument, Frew et al. found that pain, general movement, getting out, meeting friends, and financial considerations were important to patients with EB [29]. These factors are particularly important in EB where patients and carers enduring painful chronic disease with elaborate dressing changes have an important contribution to offer in terms of what is of benefit to them.

Several secondary endpoints of the EASE trial validate optional further treatment benefits. Five secondary endpoints are based on PROs, namely the change from baseline in “background pain” and “procedural pain,” in “itch,” in the impact of wounds on sleep, the evaluation of the patient’s satisfaction with treatment, and the days missed from school or work due to EB. The 2009 FDA guidance for industry “Patient-reported Outcome Measures: Use in Medical Product Development to Support Labeling Claims” [30] recommends to evaluate a PRO instrument, among others, based on the target patient population and the PRO instrument’s conceptual framework.

Mordin et al. conducted a review to evaluate health-related quality-of-life (HRQoL) measures for use in a pediatric patient population (aged 3 to < 18 years) with EB [31]. They identified 40 eligible publications and assessed them according to the 2009 FDA guidance on PRO measures and the 2005 European Medicines Agency (EMA) reflection paper on the use of HRQoL measures. In particular, they investigated the practicality of the instruments including the availability of age-appropriate version(s), the number of items (i.e. the respondent burden), and the recall period. In addition, content validity in terms of relevant content for patients with EB as well as the age relevance of concepts addressed has been assessed. Psychometric properties such as validity and reliability (test–retest, internal consistency) have been evaluated. Finally, they have checked whether the HRQoL instruments have been used in previous EB studies and whether any responsiveness to change has been observed. Mordin et al. concluded that a HRQoL instrument evaluating age-appropriate concepts for EB was not available and that content validity was lacking in the majority of measures evaluated [31].

In addition, most items of disease-specific HRQoL scores would not be changed by the treatment with an investigational medicinal product such as Oleogel-S10, as demonstrated by the studies of Lara-Corrales et al. and Venugopal et al. [22, 32]. Although wound size reductions of > 50% have been observed in both studies, neither the Dermatology Life Quality Index (DLQI)/ Children’s Dermatology Life Quality Index (CDLQI) nor the Quality of Life in Epidermolysis Bullosa (QOLEB) were able to detect significant changes [22, 32]. Similarly, treatment with Oleogel-S10 is expected to reduce the total body wound burden of EB wounds. However, this fact would not be reflected in any of the 17 items of the QOLEB, as most items rather measure disease damages and their consequences than disease activity and its impact. Hence, the concepts “pain,” “itch,” “impact of wounds on sleep,” and the patient’s satisfaction with treatment are assessed with concept-specific instruments.

### Concepts measured: pain, itch, impact of wounds on sleep, patient’s satisfaction with treatment, and days missed from school or work

For the development of the iscorEB [14], patients with EB were asked for their perception of disease severity. “Pain” and the “extent and healing of wounds” were the most common items listed by patients. Fine et al. assessed pain in children with EB and reported that only 12–14% of children with EBS, JEB, and dominant DEB and 5% of children with RDEB were pain-free [33]. Oleogel-S10 is suggested to reduce both “background” pain due to the decrease of total body wound burden and “procedural” pain because of less adherence to wound dressings. The 2009 FDA guidance states that patients from the target population might be queried about pain severity using a single-item PRO instrument to assess the efficacy of treatment on pain. Therefore, an existing single-item instrument was chosen that reliably measures “pain severity” in children enrolled in the clinical trial. In patients aged ≥ 4 years, the Wong-Baker FACES® Pain Rating Scale is used for assessing “background” pain before wound dressing change and “procedural” pain after wound dressing change. As the 2009 FDA guidance discourages proxy-reported outcome measures and recommends reports that include only those events or behaviors that can be observed instead [30], the Face, Legs, Activity, Cry, Consolability scale is used for assessing “background” pain before wound dressing change and “procedural” pain after wound dressing change in patients aged < 4 years.

Patients with EB rate itch as the most bothersome complication; 87% of patients report itch to be present at rest. Itching correlates positively with self-reported EB severity and with total body wound burden. It is strongest in healing wounds (*p* < 0.001), skin around wounds (*p* < 0.001), dry skin (*p* = 0.001), and infected wounds (*p* = 0.002) [34]. Oleogel-S10 is supposed to decrease itch by reducing the total body wound burden. Similar to the evaluation of pain severity, a single-item PRO instrument is used to assess the efficacy of treatment on itch. Patients aged ≥ 4 years and up to 13 years are asked to assess itching using the Itch Man Scale. In patients aged ≥ 14 years, itch is evaluated using the Leuven Itch Scale.

When patients with EB were asked for their perception of disease severity as basis for the development of the iscorEB, “sleep” was one of the items chosen for inclusion in the score [14]. The sleeping domain asks the patient how much sleep disturbance he/she typically experienced in the last four weeks [14]. Although this patient-derived item is part of the iscorEB, it rates sleep disturbance *per se* without relating it to, for example, the total body wound burden and its impact on sleep. The only disease-specific instrument that deliberately asks for the impact of wounds on sleep is the Wound Quality of Life Questionnaire (W-QoL) developed by Blome et al. in 2014 and based on the Freiburg Quality of Life Assessment for Wounds, the Cardiff Wound Impact Schedule, and the Würzburg Wound Score [20]. Since most of the 17 items of the W-QoL questionnaire relate to (chronic) wounds, but not to EB, the single-item PRO regarding the impact of wounds on sleep is the only W-QoL measure used in EASE.

The patient’s satisfaction with treatment is assessed with an existing PRO instrument [35]. The PRO on days missed from school or work is based on the Work Productivity and Activity Impairment Questionnaire Psoriasis, Version 2 [36].

### Patient population

The underlying genetic nature of EB is a further factor that affects clinical outcome versus established endpoints for wound-healing trials. In burns, for example, once the injuring stimulus is removed, the wound is free to heal without risk of re-injury from the original stimulus. In diabetic skin lesions, level of wounding is associated with the degree of glycemic control [37]. In EB, there are specific gene mutations that result in skin cleavage [1]; these cannot be corrected by application of a non-causal therapeutic (gene, cell, or protein therapy). Therefore, in EB, there is a constant molecular drive to re-injure, regardless of treatment, which results in a highly dynamic situation possibly unsuited to assessment of wound closure.

While the inclusivity of EASE in accepting patients with multiple types of EB (except EBS) is a strength in terms of understanding drug efficacy across EB sub-types, this also introduces significant variation in patient baseline characteristics that could have a bearing on the statistical analysis of the primary endpoint. The study also deviates from the Phase 2 design of intra-patient controls, thereby introducing more variability into the statistical outcome. Additionally, while genetic testing for accurate diagnosis of patients would be desirable, in order to enroll the required sample size, the EASE trial includes some centers where genetic testing is not standard practice. Therefore, some potential uncertainty about accuracy of clinically diagnosed sub-types remains. This has implications for any potential future trials in EB.

Sample size is a problem in rare diseases in general. The rarity of the diseases under study means that there is an ongoing problem with patient availability that can ultimately affect statistical power. This issue is frequently confounded by competition between trial recruitment programs when more than one drug developer is investigating therapeutics in the same indication. Additionally, EB families get into a routine with dressing changes [12] and can be reticent to do anything that might disrupt that. This factor can further influence the availability of patients willing to consent to non-standard-of-care therapy.

### Study design

EASE was originally conceived as an unblinded trial because of difficulties associated with formulating a suitable placebo control. In order to offset the problems associated with running an unblinded trial, an intra-patient crossover design was considered. A crossover design for this trial would also have been beneficial in terms of observing study drug activity across a greater range of wound types. However, the treatment period for each intervention (Oleogel-S10 and control) may not be long enough to enable wounds to return to baseline, as required for a design of this type. Therefore, EASE was designed with a single randomized treatment period. In the event, a placebo control was formulated and the trial was designed with standard study drug versus placebo treatment arms, thereby introducing a placebo arm that is technically not necessarily representative of standard of care. In fact, the standard of care differs so greatly from patient to patient in EB [12], that it is essentially impossible to define a homogenous standard of care arm in EASE. These issues inherent in crossover designs would apply to many wound-healing trials in lesions typical of EB.

At the time of publication, the EASE trial was recruiting participants (Additional file 1).

## Additional file

Additional file 1: SPIRIT 2013 Checklist: Recommended items to address in a clinical trial protocol and related documents. (DOC 117 kb)

## Abbreviations

AE: Adverse event
BSAP: Body surface area percentage
CDLQI: Children’s Dermatology Life Quality Index
DEB: Dystrophic epidermolysis bullosa
DEBRA: Dystrophic Epidermolysis Bullosa Research Association
DLQI: Dermatology Life Quality Index
EASE: Efficacy and safety of Oleogel-S10 in patients with EB is a Phase 3 trial of Oleogel-S10 in patients with EB
EB: Epidermolysis bullosa
EBS: Epidermolysis bullosa simplex
EMA: European Medicines Agency
FDA: Food and Drug Administration
HRQoL: Health-related quality of life
IDMC: Independent Data Monitoring Committee
JEB: Junctional epidermolysis bullosa
PRO: Patient-reported outcome
QOLEB: Quality of Life in Epidermolysis Bullosa
SCC: Squamous cell carcinoma
STSG: Split-thickness skin graft
TBSA: Total body surface area
TE: Triterpene extract
